# MARK4 Inhibited by AChE Inhibitors, Donepezil and Rivastigmine Tartrate: Insights into Alzheimer’s Disease Therapy

**DOI:** 10.3390/biom10050789

**Published:** 2020-05-20

**Authors:** Anas Shamsi, Saleha Anwar, Taj Mohammad, Mohamed F. Alajmi, Afzal Hussain, Md. Tabish Rehman, Gulam Mustafa Hasan, Asimul Islam, Md. Imtaiyaz Hassan

**Affiliations:** 1 Centre for Interdisciplinary Research in Basic Sciences, Jamia Millia Islamia, Jamia Nagar, New Delhi 110025, India; anas.shamsi18@gmail.com (A.S.); email2saleha@gmail.com (S.A.); taj144796@st.jmi.ac.in (T.M.); aislam@jmi.ac.in (A.I.); 2 Department of Pharmacognosy, College of Pharmacy, King Saud University, Riyadh 11451, Saudi Arabia; malajmii@ksu.edu.sa (M.F.A.); afzal.hussain.amu@gmail.com (A.H.); m.tabish.rehman@gmail.com (M.T.R.); 3 Department of Biochemistry, College of Medicine, Prince Sattam Bin Abdulaziz University, P.O. Box 173, Al-Kharj 11942, Saudi Arabia; mgulam@gmail.com

**Keywords:** acetylcholinesterase inhibitors, drug design and discovery, kinase inhibitors, molecular modeling and docking, isothermal titration calorimetry, Alzheimer’s disease, MARK4

## Abstract

Microtubule affinity-regulating kinase (MARK4) plays a key role in Alzheimer’s disease (AD) development as its overexpression is directly linked to increased tau phosphorylation. MARK4 is a potential drug target of AD and is thus its structural features are employed in the development of new therapeutic molecules. Donepezil (DP) and rivastigmine tartrate (RT) are acetylcholinesterase (AChE) inhibitors and are used to treat symptomatic patients of mild to moderate AD. In keeping with the therapeutic implications of DP and RT in AD, we performed binding studies of these drugs with the MARK4. Both DP and RT bound to MARK4 with a binding constant (*K*) of 10^7^ M^−1^. The temperature dependency of binding parameters revealed MARK−DP complex to be guided by static mode while MARK−RT complex to be guided by both static and dynamic quenching. Both drugs inhibited MARK4 with IC_50_ values of 5.3 μM (DP) and 6.74 μM (RT). The evaluation of associated enthalpy change (Δ*H*) and entropy change (Δ*S*) implied the complex formation to be driven by hydrogen bonding making it seemingly strong and specific. Isothermal titration calorimetry further advocated a spontaneous binding. In vitro observations were further complemented by the calculation of binding free energy by molecular docking and interactions with the functionally-important residues of the active site pocket of MARK4. This study signifies the implications of AChE inhibitors, RT, and DP in Alzheimer’s therapy targeting MARK4.

## 1. Introduction

Cellular functions of the human body are governed by protein phosphorylation and dephosphorylation by protein kinases and phosphatases [1,2]. The human genome encodes many protein kinases, among them many are considered as potential targets for drug design and development. Mutations and/or altered expression of these kinases are often associated with the cause and progression of diseases [3]. More than 25 kinase-targeting drugs have been approved by the US Food and Drug Administration (FDA) and many hundreds are still in the phase of clinical trials [4].

Microtubule affinity-regulating kinase (MARK4) is a member of Ser/Thr kinase involved in various biological functions including microtubule-dependent transport, microtubule dynamics, and cell polarity [5,6,7]. MARK4 is exploited as a potential drug target for cancer, neurodegenerative disorders, and metabolic disorders highlighting its clinical significance [8,9,10]. Many studies described the importance of MARK4 in obesity, diabetes, Alzheimer’s diseases (AD), and metastatic breast carcinomas [11,12,13]. The highest expression of MARK4 is found in the brain, kidney, and testes [14,15] and variation in its level leads to the altered Akt, mTOR [16], Wnt, and NF-κB [12] signaling and thereby causes associated diseases. The design and development of potential MARK4 inhibitors are of great significance to address cancer, diabetes, and AD therapy [17,18].

The progression of AD is linked with the hyperphosphorylated tau in the brain where the role of MARK4 has been well-established [19]. Phosphorylation of tau by MARK at the Ser^262^ site leads to detachment of tau from microtubules and consequently allows it to be phosphorylated by other kinases [20,21]. A study reported that methylene blue decreased MARK4-mediated tau phosphorylation in a dose-dependent manner in the 293T culture of *Drosophila* [22]. The overexpressed MARK4 is directly linked to defected synapses and dendritic spines [23]. Numerous studies suggested the potential of MARK4 as one of the best targets in Alzheimer’s therapy [22] as well as other neurodegenerative diseases [14,24]. Owing to its remarkable role in neurodegenerative disorders, MARK4 presents a novel therapeutic target, and hence, recently many studies have reported MARK4 inhibitors that can be used to treat MARK4-directed diseases. [19,25]. In light of all these important roles of MARK4, it is currently considered an attractive drug target especially for AD and some of the associated cancers.

Structure-based drug design is the best approach to identify bioactive leads with high specificity and affinity [26]. Exploring the interaction mechanisms of therapeutics and potential drugs with the proteins or target tissues is essential for pharmaceutical industries [27,28,29,30,31]. Studying protein−drug interaction is an essential and major step in pharmacological profiling. Drug−protein interactions are important to study as the binding of a ligand/inhibitor to protein affects its pharmacokinetics [32]. At present, acetylcholinesterase (AChE) inhibitors, rivastigmine tartrate (RT), and donepezil (DP) are in use to treat symptomatic patients of mild to moderate AD. RT is a carbamate inhibitor of AChE approved by the FDA for the treatment of mild to moderate AD in adults [33]. It improves the patient’s condition in all three major domains: cognitive function, global function, and behavior [34]. RT may prevent AD progression by preferential processing of amyloid precursor protein (APP) by α-secretase, preventing it from BACE1 [35]. DP is another AChE inhibitor, a piperidine-based reversible inhibitor, that is approved for first-line treatment of AD [36].

Post ligand binding to a protein, the structure and functionality are affected thus making it important to study drug−protein interactions. The role of MARK4 is well established in the case of AD and both RT and DP are used in AD treatment thereby providing a rationale to study the binding of these drugs with the MARK4. A detailed investigation of the binding of RT and DP with the MARK4 will be useful to understand molecular insights into the therapeutic mechanism. Such analysis could further strengthen our understanding to discover hidden targeting to improve effective therapeutic strategy.

In the present study, the binding mechanism and efficacy of DP and RT with MARK4 were investigated by spectroscopic, calorimetric, and cell-free enzyme assay complemented by molecular docking. 

## 2. Material and Methods

### 2.1. Materials

Both drugs RT and DP were purchased from Sigma-Aldrich Co. (St. Louis, MO, USA). Unless stated, all the chemicals were procured from Sigma-Aldrich Co. (St. Louis, MO, USA). Other reagents were analytical grade, procured from local suppliers. 

### 2.2. Expression and Purification of MARK4

Human MARK4 was cloned, expressed, and purified as per our published protocol [37,38]. The quality of purified protein was assessed by kinase assay and purity was checked by SDS-PAGE. MARK4 protein was confirmed with the help of Western blot using specific primary antibodies [39].

### 2.3. Kinase Assay for Enzyme Activity

The activity of MARK4 was measured using standard malachite green (BIOMOL^®^ reagent, Enzo Life Sciences) microtitre-plate assay using previously-published protocols [17,40]. MARK4 (4 μM) with increasing concentrations of ATP and assay buffer (20 mM Tris-HCl, pH 8.0, and 100 mM NaCl) were incubated for 15–20 min at 25 °C. Then, 100 μL of Biomol Green reagent was added to terminate the reaction followed by incubation for 20 min for color development. A multiplate ELISA reader was used to measure the absorbance of each well at 620 nm. 

ATPase inhibition assay of MARK4 was performed in the presence of increasing concentrations (0–20 μM) of DP and RT. Initially, MARK4 (4 μM) was pre-incubated with increasing concentrations of ligands at room temperature for 60 min in a 96-well plate. Subsequently, 200 μM of freshly-prepared ATP was mixed to the reaction mixture and incubated for 15–20 min at 25 °C. At the end of this time, BIOMOL^®^ reagent was added and kept for 15–20 min. The intensity of color was spectrophotometrically measured at 620 nm. The kinase activity of MARK4 was quantified and plotted as percent inhibition of DP and RT compared to the activity of native MARK4 considered as a reference of 100%.

### 2.4. Fluorescence Measurements

To study the binding affinity of DP and RT with MARK4, the fluorescence emission spectrum was recorded using the Jasco spectrofluorometer (FP-6200) and analyzed as per previous studies [41,42,43]. The observed fluorescence intensities were corrected for the inner filter effect [44]. Obtained data were fitted into the Stern−Volmer and modified Stern−Volmer equations to estimate Stern-Volmer constant (*K_sv_*), binding constant (*K*a), and the number of binding sites (*n*).

### 2.5. Thermodynamics of the Complex

Equation (1) (van’t Hoff equation) [45] was used to find the thermodynamic parameters such as changes in enthalpy, entropy, and Gibbs free energy associated with the reaction.

(1) ΔG=−RTLnK=ΔH−TΔS

*K* is the obtained binding constant, Δ*H* is the associated enthalpy change and Δ*G* is the associated free Gibbs energy change. Δ*S* denotes the associated entropy change with reaction, and R is the universal gas constant.

The slope of the van’t Hoff plot (Ln*K* on Y-axis against 1/*T* on the X-axis) gives the value of −Δ*H*/R, and the intercept giving the value of Δ*S*/R.

### 2.6. Isothermal Titration Calorimetry

Isothermal titration calorimetry (ITC) measurements were carried out on a VP-ITC microcalorimeter (MicroCal, Inc, GE, MicroCal, Northampton, MA, USA) at 25 °C. All the solutions were degassed in thermovac for 20 min to remove air bubbles. For sample preparation, recombinant MARK4 was extensively dialyzed in 20 mM Tris buffer, pH 8.5. The sample cell was filled with 20 µM MARK4 and the syringe was filled with 100 µM DP and 200 µM RT. We used standard protocol [46,47] to run the ITC in which the first injection was a false injection of 2 μL followed by injections of 10 μL of the ligands at a span of 260 s intervals into the sample cell. The stirring rate was maintained at 320 rpm. The obtained titration data were analyzed by Origin 8.0 software to estimate thermodynamic parameters such as stoichiometry of binding (*n*), enthalpy change (Δ*H*), an association constant (*K*_a_). 

### 2.7. Molecular Docking

Atomic coordinates of human MARK4 were taken from the Protein Data Bank (PDB ID: 5ES1, [48]) and refined through the Swiss PDB Viewer and the MGL tools [49]. Hydrogen atoms were added to the polar groups in the protein along with the Kollman charges using the Molecular Graphics Laboratory Tools. The docking was performed using AutoDock Vina with a blind search space for both DP and RT with the exhaustiveness of 8. The binding affinity and interaction of DP and RT towards MARK4 were studied while using PyMOL and Discovery Studio.

### 2.8. Statistical Analysis

All the experiments were performed in triplicate and the data obtained have been expressed in mean ± standard error of the mean (SEM).

## 3. Results

### 3.1. Fluorescence-Based Binding Studies

**Figure 1:**
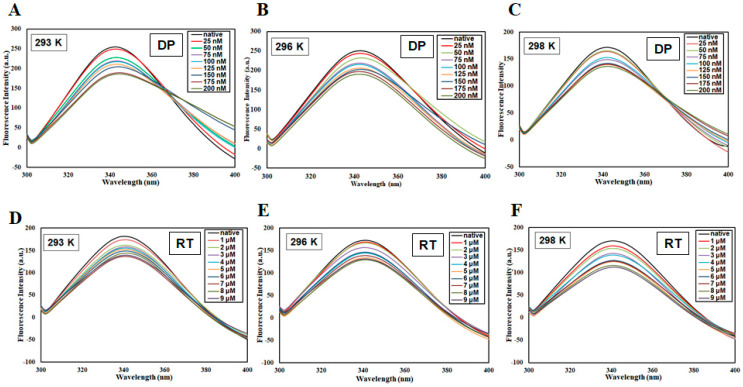
Steady-state fluorescence of MARK4 in the presence of DP (0−200 nM) at (**A**) 293 K, (**B**) 296 K, and (**C**) 298 K, and RT (0−9 µM) at (**D**) 293 K, (**E**) 296 K, and (**F**) 298 K. The concentration of MARK4 was 4 μM. The protein was excited at 280 nm and emission was recorded in the range of 300−400 nm.

The intrinsic fluorescence of protein due to tryptophan is often used to get an insight into the ligand binding to the protein because binding often leads to changes in the surrounding environment around tryptophan residues which corresponds to observed changes in fluorescence. The recombinant MARK4 was expressed and purified to carry out all binding studies [50]. Figure 1 shows the fluorescence emission spectra of MARK4 in the presence of DP (0–200 nM) and RT (0–9 µM) at three different temperatures. For both the compounds, the fluorescence intensity decreased with a subsequent increase in ligand concentrations. This decrease in fluorescence (fluorescence quenching) is indicative of a stable complex formation between protein and ligand [51]. This data was mathematically expressed to estimate associated binding parameters, Stern−Volmer constant (*K_sv_*), binding constant (*K*), and the number of the binding site (*n*). 

Fluorescence quenching can be static or dynamic or a combination of both [52,53,54]. The mode of quenching operative for a particular protein−ligand interaction can be obtained by analyzing the temperature dependency of the binding parameters since it can differentiate between static and dynamic modes of quenching [52]. Thus, to have an insight into the operative quenching mechanism, fluorescence measurements were carried out at three different temperatures. 

**Figure 2:**
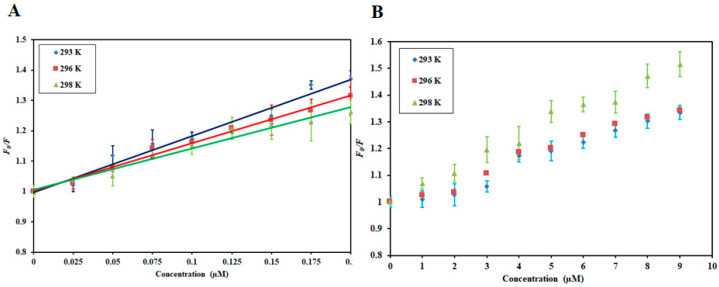
Stern−Volmer plots of (**A**) MARK4−DP and (**B**) MARK4−RT at three different temperatures. The temperatures used in the study were 293 K, 296 K, and 298 K.

Stern−Volmer (SV) plots of *F*_0_/*F* on the Y-axis and [DP] and [RT] on the X-axis is shown in Figure 2. *K_sv_* was obtained from the slope of this plot at a fixed intercept after linear regression using the Stern−Volmer equation (Equation (2)).

(2) $$\frac{F_{0}}{F}=1+K_{sv}\left[C\right]$$

The SV plot of combined quenching (both static and dynamic) is characteristically an upward curvature while the linear Stern−Volmer plot depicts that only one mode of quenching was operative. The SV plot for MARK4−DP was linear while for MARK4−RT interaction there was a slight positive deviation implying that this interaction is guided by combined quenching. The linear plot and upward curvature are not enough to define the quenching type. Hence, the variation in *K_sv_* values as a function of temperature was further analyzed. In the static quenching, there was a decrease in *K_sv_* values while in the dynamic quenching *K*_sv_ increased with a corresponding increase in temperature [55]. Table 1 depicts the *K_sv_* values obtained for MARK4−DP and MARK4−RT interactions. For MARK4−DP interaction, *K_sv_* values decrease with increasing temperature suggesting that MARK4−DP complex formation is guided by static mode of quenching. However, an opposite effect was observed for MARK4−RT interaction where an increase in *K*_sv_ values was observed with increasing temperature implying MARK4−RT interaction to be guided by a dynamic mode of quenching. Additionally, the value of the bimolecular quenching rate constant, *K_q_*, affirmed it. Equation (3) uses *K_sv_* and τ_o_ (average integral fluorescence lifetime of tryptophan) (2.7 × 10^−9^ s) [56] to find biomolecular quenching rate constant (*K*_q_)

(3) $$K_{q}=\frac{K_{sv}}{\tau_{0}}$$

The values of *K_q_* for MARK4−DP interaction and MARK4−RT interaction (Table 1) were substantially higher than the maximum dynamic quenching constant (∼10^10^ M^−1^ s^−1^) [57], suggesting that both MARK4−DP and MARK4−RT interactions are guided by static mode of quenching. For MARK4−RT interaction, earlier-obtained *K_sv_* values as a function of temperature suggested that dynamic mode guides MARK4−RT interaction. Hence, we concluded that MARK4−RT quenching is guided by a mixture of the static and dynamic modes of quenching. Figure S1 gives a clear idea of the type of quenching operative for specific protein−ligand interactions.

*K*, binding constant; *n*, number of the binding site; Δ*G*, change in Gibbs free energy; Δ*S*, change in entropy; Δ*H*, change in enthalpy.

To get insights into other binding parameters, Equation (4) was used [58]. This equation gives binding constant (*K*) and the number of binding sites (*n*).

(4) $$log\frac{F_{0}-F}{F}=logK+n\log\left[C\right]$$

**Figure 3:**
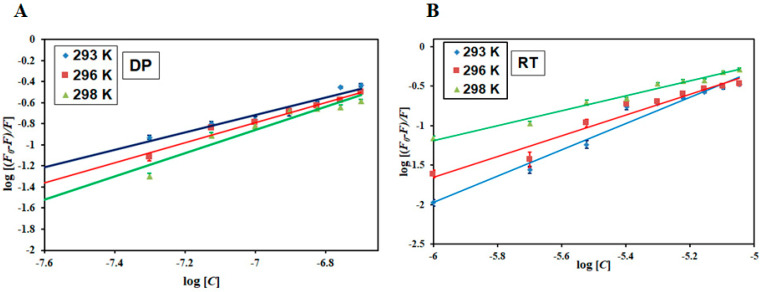
Modified Stern−Volmer plots of (**A**) MARK4−DP and (**B**) MARK4−RT at three different temperatures.

Figure 3 illustrates the experimental fitting obtained as per Equation (4) for MARK4−DP and MARK4−RT. The intercept of the plots gives the binding constant (*K*) and slope provides the number of binding sites (*n*). Table 2 lists the values of binding constants of MARK4−DP and MARK4−RT at three different temperatures. The obtained binding constants of DP and RT with the MARK4 indicate a strong affinity. The values of *K* decreased at high temperatures indicating the formation of a stable complex at a lower temperature. All these observations suggest a strong binding affinity of RT and DP to MARK4. Moreover, the MARK4−DP complex is governed by static mode and MARK4−RT complex by a combination of static and dynamic modes of quenching.

### 3.2. Thermodynamic Analysis

**Figure 4:**
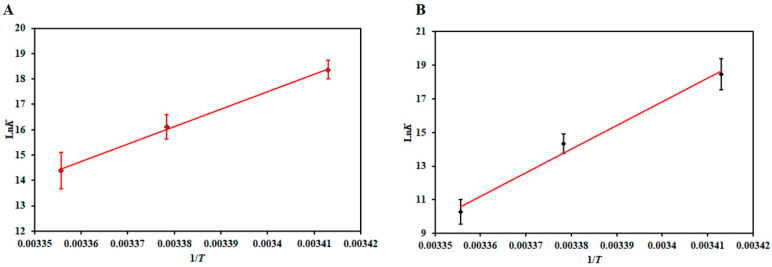
Van’t Hoff plot for (**A**) MARK4−DP and (**B**) MARK4−RT interaction at three different temperatures.

After estimating the binding parameters, the van’t Hoff equation (Equation (1)) gives the idea of different thermodynamic parameters of the complexes [52]. These values are an indicator of the prevalent driving forces associated with the binding. According to Ross and Subramaniam [59], the negative values of Δ*H* and Δ*S* suggest that this reaction is driven by van der Waals force and/or hydrogen bonding while the positive values of Δ*H* and Δ*S* suggest the reaction to be dominated by hydrophobic interactions. The negative Δ*H* and positive Δ*S* values indicate that electrostatic interactions drive the reaction [56]. Figure 4 shows the van’t Hoff plot of MARK4−DP and MARK4−RT with a linear fit of the obtained data points using Equation (1). The slope of this plot gives the value of −Δ*H*/R, and the intercept gives the value of Δ*S*/R. Table 2 lists the thermodynamic parameters estimated for MARK4−DP and MARK4−RT, respectively. The binding was spontaneous as evident from Δ*G* < 0 [60]. Moreover, the negative values of Δ*H* and Δ*S* (Table 2) obtained for both MARK4−DP and MARK4−RT suggest that the reaction is exothermic and driven by enthalpy, not entropy. The negative values of Δ*H* and Δ*S* further suggest prevalent forces driving the reactions are van der Waals and hydrogen bonding [59], thus making this process seemingly specific. 

### 3.3. Isothermal Titration Calorimetry

**Figure 5:**
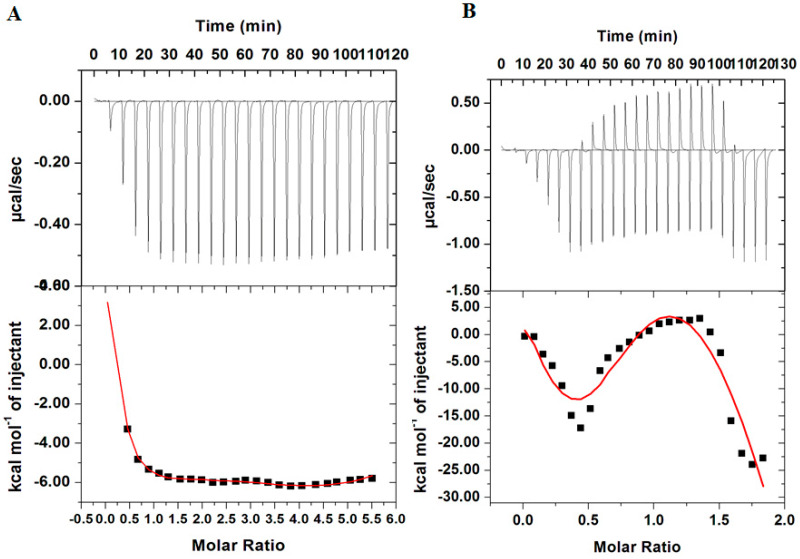
(**A**) Isothermal titration calorimetric profile obtained upon titration of 100 µM DP into 20 µM MARK4. (**B**) Isothermal titration calorimetric profile obtained upon titration of 200 µM RT into 20 µM MARK4. The bottom panel shows resultant binding isotherm before the integration of peak area and normalization to yield a plot of molar enthalpy change against the ligand/protein ratio.

To further validate the binding results obtained from fluorescence studies, ITC measurements were performed. Figure 5A shows an isotherm of MARK4 titrated with 100 µM DP while Figure 5B depicts a calorimetric profile obtained for MARK4 upon titrating with 200 µM RT. The upper panel corresponds to raw data obtained due to consecutive injection of the ligand into MARK4 with the bottom panel showing binding curves obtained after subtracting the dilution heat of both ligands and protein. The final figure was obtained using Micro Cal VP-ITC Origin 8.0., the obtained ITC isotherm advocates spontaneous binding of DP and RT with MARK4 with a significantly high binding affinity validating our fluorescence results. The thermodynamic parameters obtained for MARK4−DP interaction and MARK4−RT interaction are depicted in Table S1. Our ITC results are in agreement with fluorescence results as both methods suggested that both DP and RT have a strong binding affinity to the MARK4, despite a notable variation in the values of binding parameters being observed. These variations are due to the assumptions made in noncalorimetric approaches that Δ*H* does not depend on temperature [61,62]. Additionally, ITC measures a global change in property with fluorescence spectroscopy measuring only local changes around Tryptophan. These variations in thermodynamic parameters obtained from fluorescence spectroscopy and ITC are a common observation and has been reported in many protein−ligand interaction studies [52,56,63].

### 3.4. Enzyme Inhibition Assay

**Figure 6:**
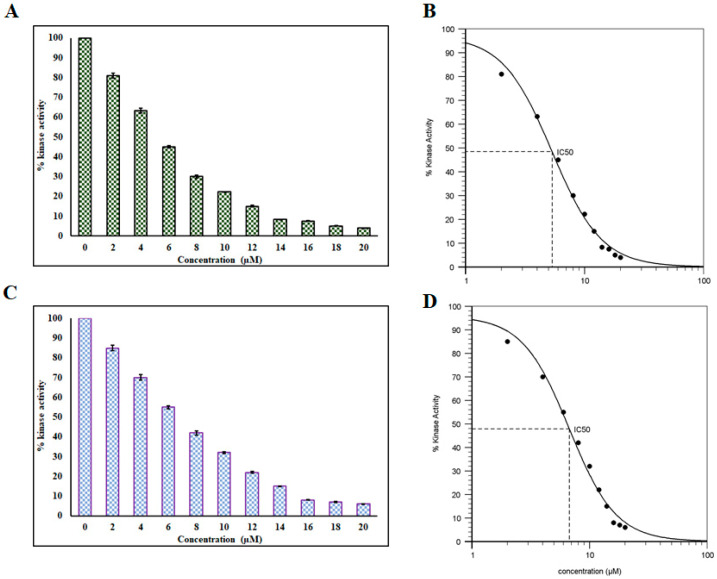
(**A**) ATPase assay showing the effect of DP on the kinase activity of MARK4. (**B**) IC_50_ plot of DP obtained through the AAT Bioquest calculator [67]. (**C**) ATPase assay depicting the effect of RT on the kinase activity of MARK4. (**D**) IC_50_ plot of RT obtained through the AAT Bioquest calculator [67].

Enzyme inhibition assays were performed to see the effect of RT and DP on the kinase activity of MARK4 as described [64,65]. Interestingly, we observed a significant reduction in the kinase activity of MARK4 with increasing concentrations of DP (Figure 6A) and RT (Figure 6C), in a dose-dependent manner. IC_50_ was calculated as 5.3 µM (Figure 6B) and 6.74 µM (Figure 6D), for DP and RT, respectively. The obtained IC_50_ values were in the order of reported inhibitors of MARK4 [6,17,66], thus implying the significance of DP and RT as potential MARK4 inhibitors. These observations suggest DP and RT have the potential to inhibit MARK4 and may be implicated in AD therapy. Finally, DP and RT bind to the MARK4 and subsequently inhibit the kinase activity of MARK4, and thus offer a newer approach of targeting AD.

### 3.5. Molecular Docking

Both DP and RT were subjected to docking analyses which showed a considerable binding affinity towards MARK4 with the calculated binding free energies as −8.1 kcal/mol and −6.3 kcal/mol, respectively. The standard compound, the co-crystalized MARK4 pyrazolopyrimidine inhibitor, 5RC (~-[(1~,6~)-6-azanyl-2,2-bis(fluoranyl)cyclohexyl]-5-ethyl-4-[6-(trifluoromethyl)pyrazolo[1 ,5-a]pyrimidin-3-yl]thiophene-2-carboxamide) was also docked to estimate the binding affinity. The binding affinity of this ligand (5RC) was calculated as −9.8 kcal/mol indicating a higher strength of binding affinity than DP and RT. The docking analysis is in agreement with our fluorescence binding studies and ITC which revealed both compounds bind to MARK4 and subsequently inhibit its kinase activity. All possible docked conformers of DP and RT were analyzed to investigate their mode of interaction with the functionally-important residues of the binding pocket of MARK4. Here, we found that DP and RT are preferentially binding with the active residues of MARK4.

**Figure 7:**
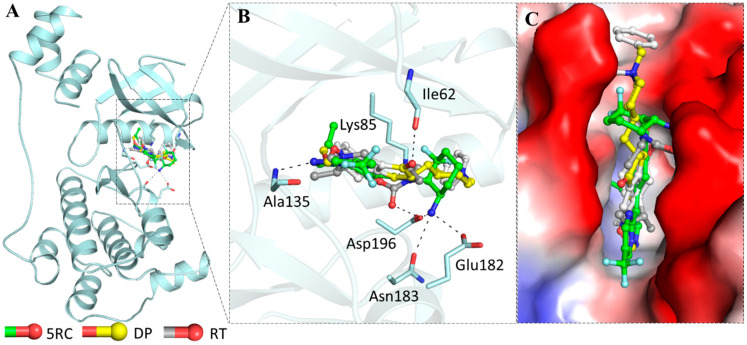
Interactions of DP and RT with MARK4. (**A**) Cartoon representation showing the docked DP, RT, and standard inhibitor 5RC, interacting with the binding site of MARK4. (**B**) Zoomed view of the binding pocket and its interaction with DP, RT, and standard MARK4 inhibitor. (**C**) Potential surface view of MARK4 binding pocket occupied by DP, RT, and standard MARK4 inhibitor.

**Figure 8:**
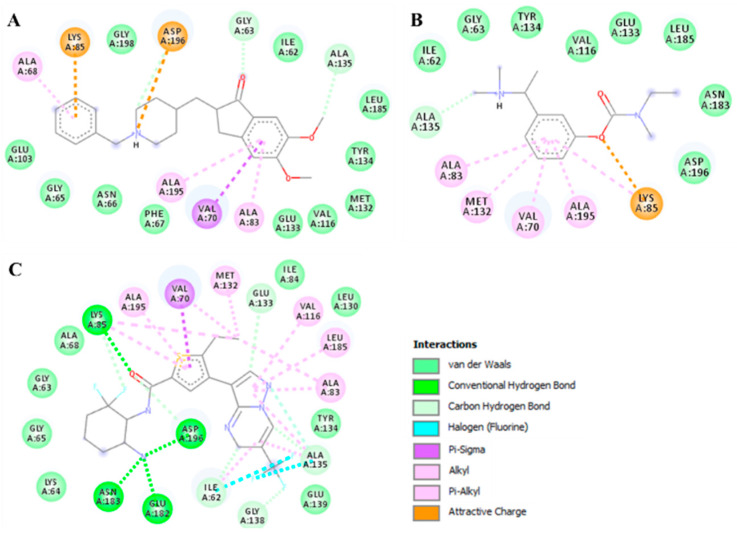
2D structural representation of MARK4 residues interacting with (**A**) DP (**B**) RT, and (**C**) standard MARK4 inhibitor (5RC, [48]).

It is well known that Lys85 is a catalytically critical residue for MARK4 kinase function and plays an important role in ATP binding which forms close interactions with DP and RT. The binding pattern of DP and RT along with the standard MARK4 inhibitor with MARK4 is illustrated in Figure 7. We observed that both DP and RT interacted with the ATP-binding site of MARK4 and mimicked the pose of a co-crystallized MARK4 inhibitor (Figure 8B). Both compounds, along with standard inhibitor [48], bind into the deep cavity of MARK4 binding pocket (Figure 8C). A set of MARK4 ATP-binding site residues is interacting with DP and RT suggesting that these compounds can block the accessibility of ATP in the binding pocket of MARK4.

Both compounds, DP and RT were further explored to see their possible interactions with the active site residues of MARK4 and to infer possible mechanisms of inhibition. It was observed that the docked complexes of MARK4 with DP and RT were stabilized by several non-covalent interactions shared by the ATP-binding site (Lys85) and other functionally-active residues of MARK4 binding pocket. The interaction pattern for DP and RT was found to be similar to the standard MARK4 inhibitor (Figure 8). It is evident from Figure 8 that DP, RT, and the standard MARK4 inhibitor are interacting with the MARK4 ATP-binding site, Lys85. Both compounds are showing common interactions and interacting with the attractive charge towards Lys85. Specific interactions between the functionally-vital residues of MARK4 binding pocket and DP and RT indicate strong binding and their further implications as ATP-competitive inhibitors of MARK4.

## 4. Conclusions

MARK4 is being used as a drug target for the development of therapeutic molecules in Alzheimer’s therapy. Overexpression of MARK4 is directly linked to the tau hyperphosphorylation which is a key event in AD pathology thus highlighting the clinical significance of developing molecules with inhibitory potential against MARK4. This study reports the inhibitory potential of AChE inhibitors, DP, and RT, against MARK4. As we know that both DP and RT are AChE inhibitors that have been recommended for the symptomatic treatment of mild to moderate AD, inhibition of MARK4 by the same drugs indicating a common targeting during AD therapy. In addition, new molecules with a high binding affinity and selectivity to MARK4 may be designed by considering the chemical scaffold of DP and RT.

## Figures and Tables

**Table 1:** Stern−Volmer constants calculated for interactions of MARK4 with DP and RT at different temperatures.

Temperature, K	*K_sv_* (10^5^ M^−1^)	*K*_q_ (10^14^ M^−1^ s^−1^)	R^2^
**MARK4−DP**			
293	18.5	6.85	0.99
296	15.7	5.81	0.98
298	13.6	5.03	0.96
**MARK4−RT**			
**Temperature, K**	***K_sv_*** **(10^4^ M^−1^)**	***K*** **_q_** **(10^13^ M^−1^ s^−1^)**	**R^2^**
293	3.4	1.25	0.97
296	3.9	1.44	0.97
298	5.8	2.14	0.98

***K_sv_***, Stern−Volmer constant; ***K*_q_**, quenching rate constant.

**Table 2:** Thermodynamic parameters obtained from fluorescence quenching studies carried out at different temperatures.

Temperature (K)	*K* 10^7^ M^−1^	*n*	Δ*G* kcal mol^−1^	Δ*S* cal mol^−1^K^−1^	Δ*H* kcal mol^−1^	TΔS kcal mol^−1^
**MARK4** **−** **DP**						
293	9.4	1.66	−10.70	−432.749	−137.5	−126.79
296	0.9	1.311	−9.40			−128.09
298	0.002	0.1	−8.54			−128.95
**MARK4−R** **Τ**						
293	10.5	1.66	−10.74	−455.78	−144.23	−133.48
296	0.16	1.311	−8.46			−135.76
298	0.002	0.94	−6.18			−138.04

## Supplementary Materials

The following can be found at https://www.mdpi.com/2218-273X/10/5/789/s1; Figure S1: Graphical representation of the operative mode of quenching for specific protein-ligand interaction; Table S1: Thermodynamic parameters obtained from ITC measurements.
