# Supplementary material for: MARK4 Inhibited by AChE Inhibitors, Donepezil and Rivastigmine Tartrate: Insights into Alzheimer’s Disease Therapy
Source: Biomolecules. 2020 May 20;10(5):789. doi: 10.3390/biom10050789 (PMC7277793; doi:10.3390/biom10050789)
Supplement: Supplementary file 1 [file biomolecules-10-00789-s001.pdf]

biomolecules

Supplementary Materials

MARK4 Inhibited by AChE Inhibitors, Donepezil and Rivastigmine Tartrate: Insights into Alzheimer's Disease Therapy

Anas Shamsi [1], Saleha Anwar [1], Taj Mohammad [1], Mohamed F. Alajmi [2], Afzal Hussain [2], Md. Tabish Rehman [2], Gulam Mustafa Hasan [3], Asimul Islam [1] and Md. Imtaiyaz Hassan [1,*]

[1] Centre for Interdisciplinary Research in Basic Sciences, Jamia Millia Islamia, Jamia Nagar, New Delhi 110025, India; anas.shamsi18@gmail.com (A.S.); email2saleha@gmail.com (S.A.); taj144796@st.jmi.ac.in (T.M.); aislam@jmi.ac.in (A.I.)

[2] Department of Pharmacognosy, College of Pharmacy, King Saud University, Riyadh, 11451 Saudi Arabia; malajmii@ksu.edu.sa (M.F.A.); afzal.hussain.amu@gmail.com (A.H.); m.tabish.rehman@gmail.com (M.T.R.)

[3] Department of Biochemistry, College of Medicine, Prince Sattam Bin Abdulaziz University, P.O. Box 173, Al-Kharj 11942, Saudi Arabia; mgulam@gmail.com

* Correspondence: mihassan@jmi.ac.in; Tel.: +91-9990323217

Received: 23 April 2020; Accepted: 17 May 2020; Published: 20 May 2020

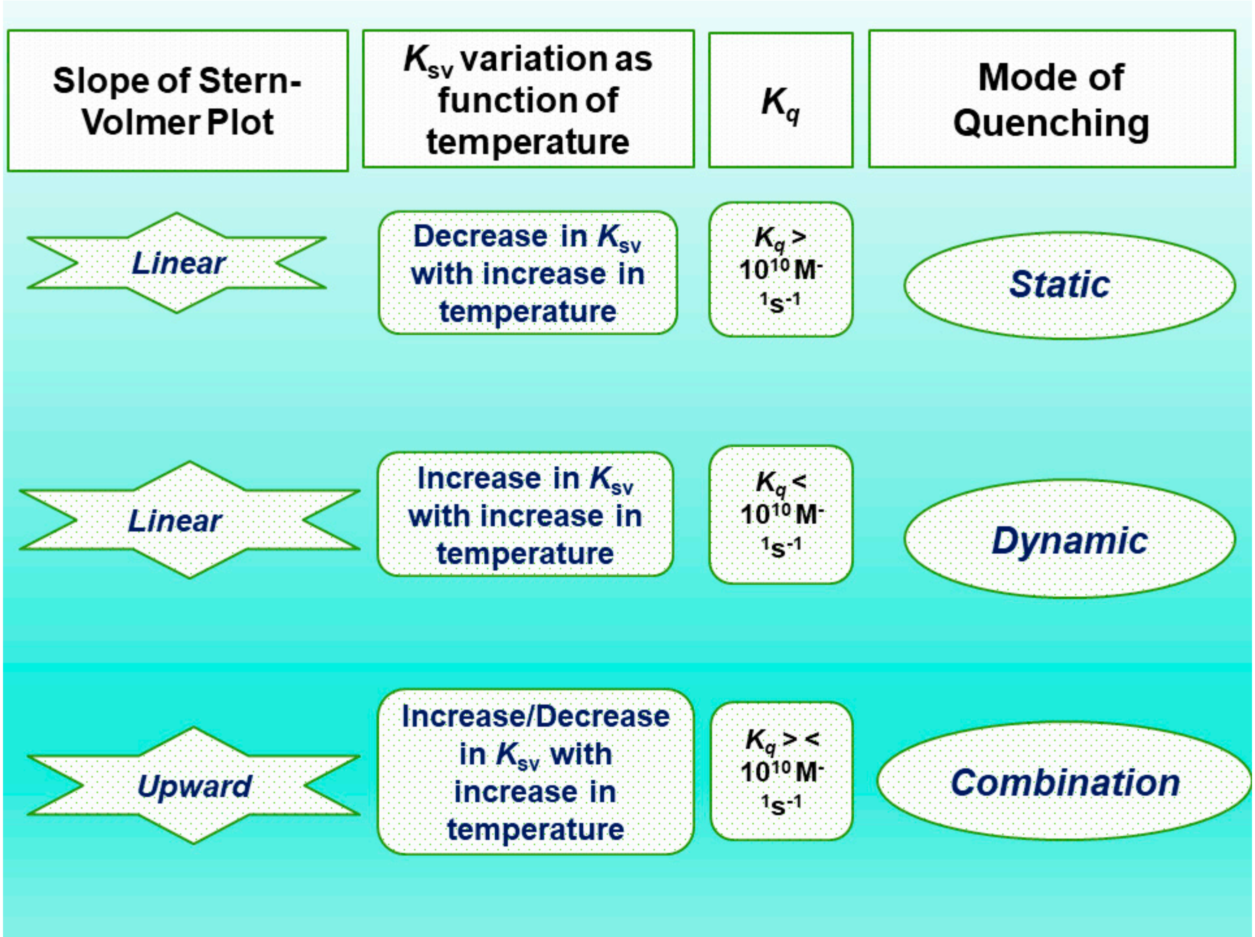

Figure S1. Graphical representation of the operative mode of quenching for specific protein-ligand interaction.

Table S1. Thermodynamic parameters obtained from ITC measurements.

MARK4-DP		
**K_a (association constant), M^{-1}**	**ΔH (enthalpy change), cal/mol**	**ΔS (cal/mol/deg)**
K_{a1}= 6.79 X 10^5 ± 2.4 X 10^5	ΔH_1= 8444 ±1.82 X 10^3	ΔS_1= 51.2
K_{a2}= 1. X 10^5 ± 1.9 X 10^3	ΔH_2= -6.88 X 10^4 ± 5.48 X 10^3	ΔS_2= -207
K_{a3}= 9.37 X 10^4 ± 3.0 X 10^3	ΔH_3= 1.01 X 10^5 ±7.49 X 10^3	ΔS_3= 368
K_{a3}= -1.044 X 10^5 ± 4.68 X 10^3 M	ΔH_4= -4.91 X 10^6 ±5.6 X 10^6	ΔS_4= -327
**MARK4-RT**		
**K_a (association constant), M^{-1}**	**ΔH (enthalpy change), cal/mol**	**ΔS (cal/mol/deg)**
K_{a1}= 1.02 X 10^5 ± 2.8 X 10^5	ΔH_1= 1329 ± 3.05 X 10^3	ΔS_1= 31.1
K_{a2}= 1.17 X 10^5 ± 7.3 X 10^4	ΔH_2= -2.15 X 10^5 ± 9.07 X 10^4	ΔS_2= -700
K_{a3}= 2.00 X 10^5 ± 1.6 X 10^5	ΔH_3= 7.86 X 10^5 ± 2.26 X 10^5	ΔS_3= 2.66 X 10^3
K_{a3}= 5.44 X 10^4 ± 2.54 X 10	ΔH_4= 2.23 X 10^6 ± 4.8 X 10^5	ΔS_4= -7.47 X 10^3
